# Investigating women’s health issues and help-seeking intentions in primary care in Japan: a cross-sectional study

**DOI:** 10.1186/s12875-022-01862-0

**Published:** 2022-09-26

**Authors:** Keiichiro Narumoto, Kei Miyazaki, Machiko Inoue, Makoto Kaneko, Tadao Okada, Motoi Sugimura

**Affiliations:** 1 Department of Obstetrics, Gynecology and Family Medicine, Hamamatsu University School of Medicine, 1-20-1 Higashiku, Hamamatsu, Shizuoka, 431-3125 Japan; 2 Morimachi Family Medicine Clinic, 387 Kusagaya, Morimachi, Shuchigun, Shizuoka, 437-0214 Japan; 3 Hamamatsu University School of Medicine Hospital General Medical Training Program / Shizuoka Family Medicine Training Program, 1-20-1 Higashiku, Hamamatsu, Shizuoka, 431-3125 Japan; 4 Department of Education for Community-Oriented Medicine, Nagoya University Graduate School of Medicine, 65 Tsuruma-cho, Showa-ku, Nagoya, Aichi 466-8550 Japan; 5 Department of Family and Community Medicine, Hamamatsu University School of Medicine, 1-20-1 Higashiku, Hamamatsu, Shizuoka, 431-3125 Japan; 6 Department of Health Data Science, Yokohama City University, 22-2 Seto, Kanazawaku, Yokohama, Kanagawa 236-0027 Japan; 7 Tessyoukai Kameda Family Clinic Tateyama, 4304-9, Masaki, Tateyamashi, Chiba, 294-0051 Japan

**Keywords:** Women’s health care, Primary care, Help-seeking intention

## Abstract

**Background:**

Many women face a variety of barriers to seeing obstetricians and gynecologists (OB/GYNs). Primary care physicians (PCPs) in Japan are not well equipped to address and adequately handle women’s health issues. Hence, opportunities for women to consult PCPs about women’s health issues are often limited during busy outpatient encounters. It is essential to explore PCP’s roles in women’s health care by examining women’s health needs in a primary care setting. The aim of the study is to describe the prevalence and distribution of women’s health issues and help-seeking intentions among women visiting a primary care clinic.

**Methods:**

This was a cross-sectional study using a questionnaire. We included women aged 20–60 years who visited a primary care clinic for any reason. The questionnaire comprised a list of women’s health issues, the General Help Seeking Questionnaire to assess help-seeking intentions, and participants’ demographics including their reasons for visiting and regularity of OB/GYN visits.

**Results:**

We distributed the questionnaire to 294 women and analyzed 260 valid responses. The average age of the respondents was 40.5 years old, and they had an average of 5.2 clinic visits a year. Approximately half of them (50.4%) visited for their own care. One hundred thirty-nine (53.9%) reported at least one women’s health issue, and 73.9% of them had no regular visit to an OB/GYN. The major concerns of women’s health issues included gynecological cancer screenings and menstrual problems. The distribution of help-seeking intentions for each source of care appeared to be classified into three patterns. One fifth of the women indicated high help-seeking intentions for PCPs, and a greater number of women expressed higher help-seeking intentions for PCPs when they did not regularly see an OB/GYN.

**Conclusions:**

A significant number of women who visited a primary care clinic had a specific concern about women’s health issues, and a majority of them had not regularly visited their OB/GYN. PCPs may have an important role in providing an opportunity for women to discuss their concerns about women’s health issues as part of comprehensive care during a daily clinical encounter.

## Background

Comprehensive care for women across life stages is increasingly emphasized, but actual care remains fragmented [1–4]. In Japan, women’s health has conventionally been handled by obstetricians and gynecologists (OB/GYNs). However, they typically do not pay much attention to comprehensive care beyond active health problems [5]. The primary health care system in Japan is characterized by universal health insurance and free access, and primary care is provided by different specialties at any levels of medical facilities [6]. Primary care providers receive reimbursement primarily from tests and prescriptions under a uniform national fee schedule, not from counseling or complex, comprehensive medical consultation. Therefore, the nature of outpatient encounters is often problem-focused and short in time, approximately 6–10 minutes on average [6]. Infrastructure of primary care remains under development, and the number of physicians certified in family medicine by the Japan Primary Care Association (JPCA), the professional body representing primary care, is very small, 794 as of April 15, 2022 [7].

Women visit primary care more often than they visit hospital outpatient clinics [8] and many are reluctant to visit OB/GYNs [9, 10]. Hence, primary care physicians (PCPs) play an important role in delivering comprehensive, well-coordinated and preventive women’s health care. However, PCPs in Japan face challenges in doing so. They have very limited training opportunities for women’s health [11] and many of them feel they lack knowledge and skills in obstetrics and gynecology [12]. In daily practice, they may make conscious decisions about whether to facilitate women’s disclosure of women’s health issues during a patient encounter [13]. They may wonder if asking and talking about women’ health issues is irrelevant or inappropriate, even as part of comprehensive care, depending on the reason for their visit, the patient-doctor relationship, and the dialogue during their visits. From women’s perspectives, they may be hesitant to voluntarily discuss women’s health issues with PCPs for a variety of reasons [14]: concerns about unnecessary visits or bothering a physician, wasting the time, unpleasant experiences related to health services providers, and fear of the consequences of medical help-seeking. They may wonder if PCPs are the right people to ask about women’s health issues under the specialized medical system in Japan.

What are women’s health needs in primary care in Japan? To what extent are women willing to consult PCPs about their women’s health issues? Investigating women’s health issues and help-seeking behaviors, particularly among women who visit primary care for health concerns other than women’s health issues, could further expand the discussion about PCPs’ roles in women’s health care in Japan. This study aims to describe the prevalence and distribution of women’s health issues and help-seeking intentions among women in primary care.

## Methods

In this article, we report the results of a cross-sectional data analysis from mixed methods research that we conducted to examine help-seeking behaviors for women’s health issues and patient experience in primary care. The study was approved by the Clinical Research Ethics Committee of Hamamatsu University School of Medicine on March 29, 2019 (19–027).

### Setting

We conducted the study in four primary care clinics (A, B, C, and D) from April to September 2019. Clinics A, B, and C are public facilities in rural areas with a population of approximately 20,000-50,000, while D is a private facility in an urban area with a population of approximately 1.5 million. All facilities offer group practice with family physicians certified by JPCA and family medicine residents. The former three facilities provide primary care in obstetrics and gynecology, where some of the certified family physicians who have a special interest and have trained in obstetrics and gynecology handle common gynecologic problems and provide low-risk prenatal care as well as perform transvaginal ultrasounds if medically indicated. In our study, we defined PCPs as physicians specializing in or training in family medicine, excluding OB/GYNs, pediatricians or internists.

### Participants

We recruited women between the ages of 20 and 60 years who presented to any of the four clinics regardless of their reasons for visiting and who gave written consent to participate in the study. We employed the consecutive sampling method, where a female researcher spent three to 4 days at each clinic during the study period and approached all the study candidates. Since family care is one of the important roles of primary care, women who accompanied their family members to the clinics were also included. We excluded those who had difficulty understanding the study or reading and writing Japanese. We gave a 300 Japanese yen gift card to the study participants as an honorarium when they completed the questionnaire.

### Measures

#### Questionnaire

With consent for study participation, we asked the women to answer the questionnaire in a waiting room before their medical consultation. The questionnaire items comprised the reason for visit, number of annual clinic visits, a list of women’s health issues, help-seeking intentions for women’s health issues, past cervical and breast cancer screening tests, regularity of visit to OB/GYN, and the patient’s demographics.

Women’s health issues included the following health problems commonly encountered in primary care: dysmenorrhea, hypermenorrhea, menstrual irregularity, uterine prolapse, abnormal vaginal discharge, abnormal uterine bleeding, diseases of the uterus and ovaries (e.g., uterine myoma, ovarian tumor), postmenopausal symptoms, sexual dysfunction and issues related to sexuality (e.g., sexual orientation/gender identity), genital symptoms, contraception, breast-related issues (e.g., lump, abnormal nipple discharge, breastfeeding), pregnancy and infertility, and gynecological cancer screening. Plain language explanations were added to each item of women’s health issues to encourage the participant’s understanding.

The participants were asked to answer their help-seeking intentions only when they reported at least one women’s health issue. The help-seeking intentions were measured with reference to the General Help-Seeking Questionnaire [15], which is a commonly used questionnaire in the absence of a gold standard for measuring help-seeking behaviors [16]. The participants rated how likely they would consult, on a seven-point Likert scale (1: least likely, 7: most likely), with each of the following sources of care: PCPs at the aforementioned clinics, OB/GYNs, public health nurses, nurses/midwives, pharmacists, acupuncturists/chiropractors, family/close friends, media (internet, book, magazine) and others.

The Ministry of Health, Labour and Welfare recommends that cervical and breast cancer screening be performed once every 2 years [17], so we asked whether they had received a Papanicolaou test and mammography within the past 2 years. A regular visit to the OB/GYN was defined as having visited any OB/GYN clinic in the past year.

Participant demographics included age, self-rated health, self-rated economic conditions, highest level of education completed, and health literacy. Self-rated health and economic conditions were assessed on a five-point Likert scale. Health literacy was assessed on a five-point Likert scale for a total of five questions on communicative and critical health literacy [18].

#### Outcomes

For the aims of the study, the outcomes were the prevalence and distribution of women’s health issues and the patterns of help-seeking intentions for women’s health issues .

#### Analysis

Since our research interest was women’s health issues and help-seeking intentions among women visiting for health concerns other than women’s health issues, we excluded those who presented to the clinics mainly for women’s health issues from analysis. We mainly employed descriptive analysis. For differences in the demographics of the participants with or without women’s health issues, we conducted chi-square test for nominal variables and independent t-test for continuous variables. In the quantitative study within the mixed methods research that we conducted, we employed logistic regression analysis to examine a relationship between help-seeking intentions from PCPs (dependent variable) and patient experience of primary care attributes (independent variable) among women with at least one women’s health issue. Help-seeking intentions were classified as high or low after the median. Since the number of events per variable values of 10 or greater was necessary for logistic regression analysis [19] and the maximum number of independent variables was nine in the study, we calculated a minimum sample size of 90 per event. Given that a half of women with women’s health issues have a higher help-seeking intention and the expected prevalence of women’s health issues of up to 75% from the existing studies [20, 21], we estimated that a sample size of 240 would be necessary.

## Results

**Fig. 1:**
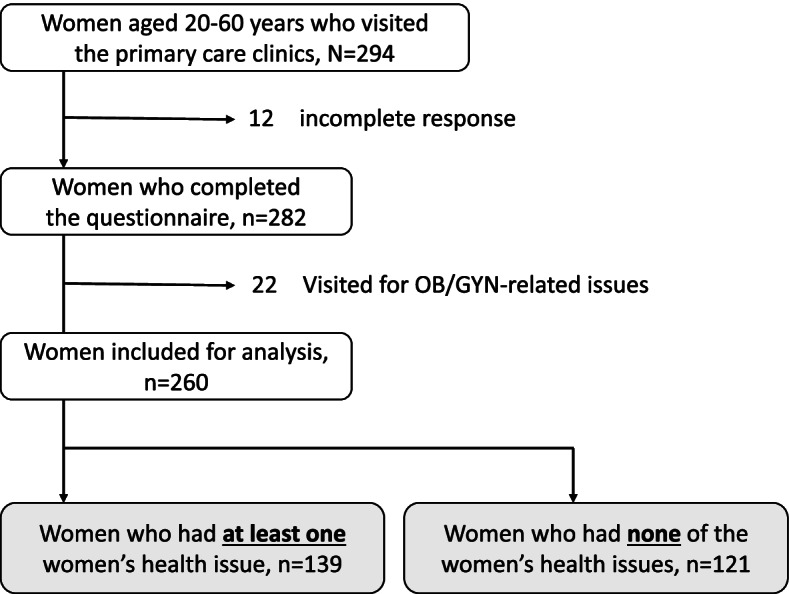
Selection of the participants for analysis. The questionnaire was distributed to 294 women who visited the primary care clinics during the study period. Twelve responses were excluded for incomplete answers, resulting in 282 valid responses. For our research purpose, we excluded 22 responses because those responders visited a primary care clinic for women’s health issues, and then conducted an analysis for a total of 260 responses

The questionnaire was distributed to a total of 294 women who visited the primary care clinics. We excluded 12 responses for incomplete answers and 22 responses of women whose reasons for their visits were women’s health issues, which resulted in 260 valid responses for analysis (Fig. 1).

Table 1 shows the demographics of the participants. The numbers (%) of participants from each clinic (A, B, C, D) were 93 (36), 44 (17), 40 (15) and 83 (32), respectively. Approximately half of all respondents visited to seek their own care on the day of the survey. The majority of reasons for clinic visits were chronic diseases (e.g., hypertension, dyslipidemia, diabetes) followed by acute illnesses (e.g., upper respiratory infections, headache, acute low back pain), laboratory tests and preventive care (e.g., immunization, health check-up). The average age of the respondents was 40.5 years old, and a majority of them (97%) had at least graduated from high school. They had an average of 5.2 clinic visits per year, and most of them had updated cervical and breast cancer screening tests within the past 2 years (68 and 69%, respectively). Approximately three-quarters of them (74%) had not been to an OB/GYN in the past year.

**Table 1:** Demographics of the participants (*n* = 260)

Characteristic	All *N* = 260	Women’s health issues^a^		
		At least one (*n* = 139)	None (*n* = 121)	*P* Value^b^
The numbers of reported women’s health issues, n (%)				
1		57 (41)		
2		41 (30)		
3		18 (13)		
4		9 (7)		
5		7 (4)		
6		4 (3)		
7		3 (2)		
Reason for visit, n (%)				
Self-referral	131 (50)	70 (50)	61 (50)	.993
Accompanying family members	129 (50)	69 (50)	60 (50)	
Primary care clinic, n (%)				.323
A	93 (36)	46 (33)	47 (39)	
B	44 (17)	25 (18)	19 (16)	
C	40 (15)	18 (13)	22 (18)	
D	83 (32)	50 (36)	33 (27)	
Age, yrs. (SD)	40.5 (10.1)	39.8 (9.3)	41.4 (10.9)	.195
Frequency of clinic visit, times/year (SD)	5.2 (4.8)	5.3 (4.5)	5.2 (5.2)	.889
Missing	3	1	2	
Self-rated health, n (%)				.064
Good	37 (14)	13 (9)	24 (20)	
Somewhat good	86 (33)	43 (31)	43 (36)	
Average	88 (34)	51 (37)	37 (31)	
Not so good	44 (17)	29 (21)	15 (12)	
Not good	4 (2)	2 (1)	2 (2)	
Missing	1	1		
Self-rated economic conditions, n (%)				.028
Very comfortable	12 (5)	7 (5)	5 (4)	
Moderately comfortable	45 (17)	27 (19)	18 (15)	
Average	154 (59)	70 (50)	84 (69)	
Somewhat struggling	43 (17)	30 (22)	13 (11)	
Greatly struggling	5 (2)	4 (3)	1 (1)	
Missing	1	1		
Highest level of education completed, n (%)				.294
Junior high school	8 (3)	2 (1)	6 (5)	
High school	90 (35)	45 (33)	45 (37)	
Vocational school or junior college	94 (36)	54 (39)	40 (33)	
University or graduate school	67 (26)	37 (27)	30 (25)	
Missing	1	1		
Cervical cancer screening within the past 2 years, n (%)				.659
Yes	177 (68)	94 (68)	83 (69)	
No	55 (21)	28 (20)	27 (23)	
Never	26 (10)	16 (12)	10 (8)	
Missing	2	1	1	
Breast cancer screening within the past 2 years, n (%)^c^				.243
Yes	92 (69)	50 (71)	42 (67)	
No	28 (21)	16 (23)	12 (19)	
Never	13 (10)	4 (6)	9 (14)	
Regular visit for OB/GYN clinic^d^, n (%)				.812
Yes	66 (25)	36 (26)	30 (25)	
No	193 (74)	102 (74)	91 (75)	
Missing	1	1		
HL^e^, mean (SD) score	3.86 (0.58)	3.94 (0.48)	3.78 (0.67)	.030
Missing	5	2	3	
Communicative HL	4.05 (0.70)	4.14 (0.57)	3.94 (0.82)	.023
Missing	4	2	2	
Critical HL	3.60 (0.72)	3.63 (0.71)	3.55 (0.73)	.348
Missing	2	1	1	

*OB/GYN* Obstetrics/Gynecology, *HL* Health literacy, *SD* Standard deviation

^a^The presence of women’s health issues is defined as having at least one concern about dysmenorrhea, hypermenorrhea, menstrual irregularity, uterine prolapse, abnormal vaginal discharge, abnormal uterine bleeding, diseases related to the uterus or/and ovaries, menopausal symptoms, sex-related issues including sexual orientation/gender identity and sexual dysfunction, genital symptoms, contraception, breast-related issues including breast lumps, abnormal nipple discharge or/and breastfeeding, pregnancy or infertility, or gynecologic cancer

^b^By chi-square test for nominal variables and independent t-test for continuous variables

^c^Only for those aged 40 and older (*n* = 133)

^d^Defined as having been to obstetrics/gynecology specialists within the past year

^e^Assessed using the instrumental tool by Ishikawa et al. [18]

Table 1 shows the demographics of the participants. Approximately half of all respondents visited to seek their own care. The average age of the respondents was 40.5 years old, and they had an average of 5.2 clinic visits a year. A majority of them had graduated from high school (96.5%) and had updated cervical and breast cancer screening tests within the past 2 years (68 and 69%, respectively). Approximately three-quarters of them (74%) had not been to an OB/GYN in the past year. One hundred thirty-nine (54%) reported at least one women’s health issue. The number of women was highest at one women’s health issue and lowest at seven women’s health issues, revealing a descending trend. There were similarities observed in age, frequency of clinic visit, cancer screening status and regular visit for OB/GYN clinic between those who had at least one women’s health issue and those who reported none. The participants with women’s health issues tended to have a higher score on health literacy, while those without women’s health issues tended to report better self-rated health and give more neutral responses to self-rated economic conditions

One hundred thirty-nine (54%) reported at least one women’s health issue. The proportion of women reporting women’s health issues was similar between those self-referring (50.4%) and those accompanying their family members (49.6%). Approximately three-quarters (74%) of them had no regular visit to an OB/GYN. There were similarities observed in age, frequency of clinic visit, cancer screening status and regular visit for OB/GYN clinic between those who had at least one women’s health issue and those who reported none. The participants with women’s health issues tended to have a higher score on health literacy, while those without women’s health issues tended to report better self-rated health and give more neutral responses to self-rated economic conditions.

**Fig. 2:**
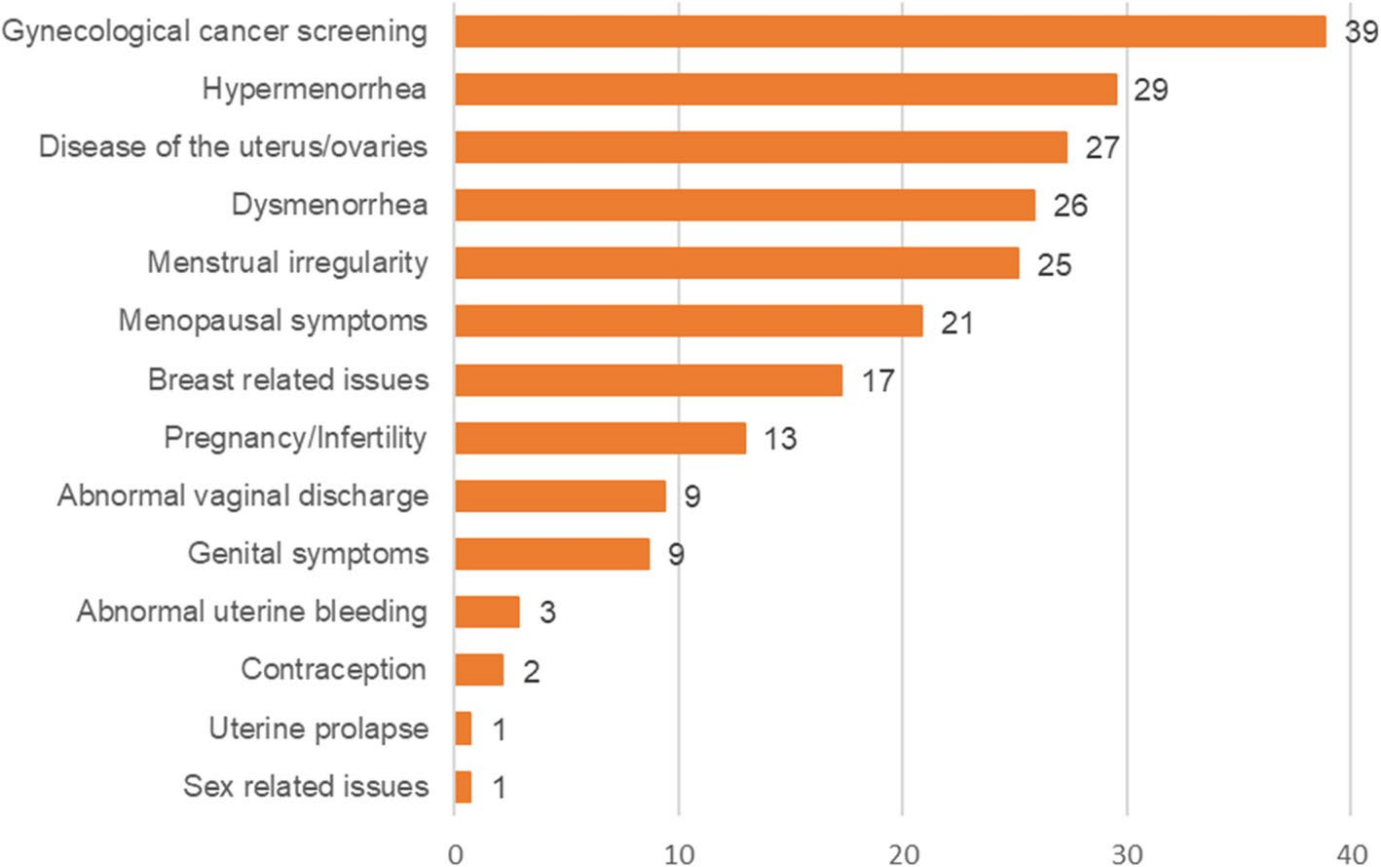
Self-reported women’s health issues of the participants (*n* = 139, multiple answers allowed). Figure 2 reveals the distribution of each category of women’s health issues that the participants (*n* = 139) reported. Concern about gynecological cancer screening tests was the most frequently reported (39%). Menstruation-related issues, such as hypermenorrhea (30%), dysmenorrhea (26%) and menstrual irregularity (25%), occupied the top five places. A specific disease of the uterus or ovaries (27%) and menopausal symptoms (21%) were the third and fifth concerns from the top, respectively

Figure 2 reveals the distribution of each category of women’s health issues that the participants (*n* = 139) reported. Concern about gynecological cancer screening tests was the most frequently reported (39%). Menstruation-related issues, such as hypermenorrhea (30%), dysmenorrhea (26%) and menstrual irregularity (25%), occupied the top five places. A specific disease of the uterus or ovaries (27%) and menopausal symptoms (21%) were third and fifth concerns from the top, respectively.

**Fig. 3:**
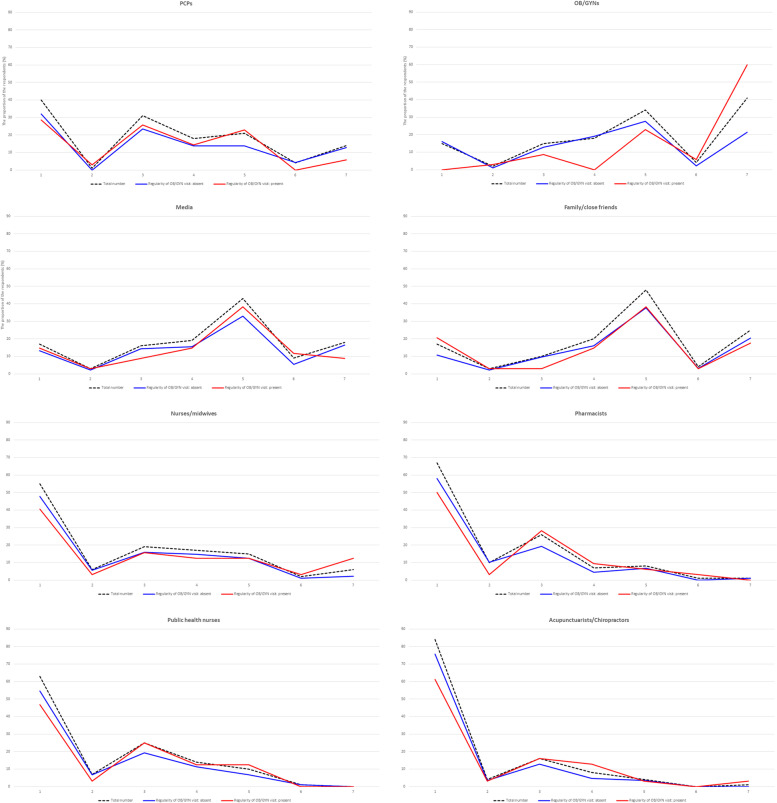
Distribution of help-seeking intentions by regularity of OB/GYN visits. Figure 3 shows the distribution of the help-seeking intention levels for each source of care. The X-axis indicates the help-seeking intention level ranging from 1 of “least likely” to 7 of “most likely.” The Y-axis designates both the total number of women by help-seeking intention level (black dotted line) and the proportion of women in each help-seeking intention level according to whether they had regular visits to their OB/GYN (red line) or not (blue line). The overall distributions (black dotted line) of help-seeking intentions seemed to be classified into three patterns. For PCPs, acupuncturists/chiropractors, nurses/midwives, public health nurses and pharmacists, there was a descending trend in the number of respondents as the help-seeking intention level increased. An opposite trend was observed for OB/GYN. For media and family/close friends, the frequency peaked at a relatively high score of five. A higher number of women showed a higher level of help-seeking intention for PCPs, that is, six or seven on the scale, when they did not have regular visits to their OB/GYN (blue line). This trend was similarly observed in media. On the other hand, a greater number of women indicated the highest level of help-seeking intention for OB/GYN and nurse/midwife when they had regular visits to their OB/GYN (red line)

Figure 3 shows the distribution of the help-seeking intention levels for each source of care. The overall distributions of help-seeking intentions seemed to be classified into three patterns. For PCPs, acupuncturists/chiropractors, nurses/midwives, public health nurses and pharmacists, there was a descending trend in the number of respondents as the help-seeking intention level increased. An opposite trend was observed for OB/GYN. For family/close friends and media including internet, books or magazines, the frequency peaked at a relatively high score of five. A higher number of women showed a higher level of help-seeking intention for PCPs, that is, six or seven on the scale, when they did not have regular visits to their OB/GYN. This trend was similarly observed in media. On the other hand, a greater number of women indicated the highest level of help-seeking intention for OB/GYN and nurse/midwife when they had regular visits to their OB/GYN.

The participants who showed a help-seeking intention level of five or higher had 20% for PCPs, approximately 55% for OB/GYNs, family/close friends and media, and less than 10% for nurses/midwives, public health nurses, pharmacists and acupuncturists/chiropractors.

## Discussion

This study is the first to our knowledge to investigate women’s health issues and help-seeking intentions among women who visit a primary care clinic in Japan. It is worth noting that approximately half of the participants reported some kind of women’s health issue, and approximately 70% of them had not visited an OB/GYN in the past year. It is also unique in that the study included women who accompanied their family members from the perspective of family care, and they also had approximately 50% reporting at least one women’s health issue. Furthermore, one-fifth of women with women’s health issues indicated a high likelihood of seeking help from PCPs.

These findings highlighted that women’s health issues need to be addressed in primary care in Japan and that PCPs must be well equipped with skills to adequately handle women’s health issues when appropriate. PCPs may be required to proactively tap into women’s concerns about women’s health issues during a clinical encounter. When initiating a conversation about women’s health issues, it seems more satisfying to women if PCPs address a broad context of health-related questions [22]. Additionally, PCPs may need to recognize that routine physical examination and medical conditions other than women’s health issues were the most common reasons for visit when women’s health issues were initially brought up in the previous study [23], which may help reduce the psychological burden on PCPs who feel discussing women’s health issues in their daily practice is irrelevant.

Gynecological cancer screening and menstrual problems were the top concerns in the study. Our results on the prevalence of dysmenorrhea, menstrual irregularity and hypermenorrhea were similar to those in the previous larger cross-sectional studies in Japan. An online survey of approximately 20,000 Japanese women aged 15–49 years revealed 74% of them had menstrual problems and 50 and 19% of them had dysmenorrhea and hypermenorrhea, respectively [20]. Another survey of over 2000 Japanese female workers showed 78% of them had some degree of menstrual pain and 17 and 28% of them had menstrual irregularity and severe dysmenorrhea, respectively [21]. Despite the differences in the target population and the definitions of menstrual problems as well as slight fluctuations in the prevalence rates, a significant number of women suffer from menstrual problems. It is essential to take and interpret a history of menstruation appropriately and determine whether the situation merits a gynecological exam, treatment or referral to an OB/GYN. For gynecological cancer screening, PCPs need to clarify what women’s concerns are and appropriately respond to them. If women are concerned about their family history of breast, ovarian and uterine cancer, PCPs require skills in properly collecting a detailed family history and providing genetic counseling as needed. PCPs should acquire adequate knowledge and examination skills to integrate a variety of aspects of women’s health care into their daily practice in an effective way.

In the previous ecology and cross-sectional studies in other countries [23–25], the number of women who self-referred to a gynecologist for women’s health issues was 1.5 to 13 times higher than that of PCPs. However, neither study specifically targeted women who visited a primary care clinic or visualized and analyzed the patterns of help-seeking intentions across different sources of care, considering whether women regularly visited an OB/GYN. Our study results provided intriguing insights into women’s help-seeking intentions for women’s health issues. A greater number of women expressed higher help-seeking intentions to PCPs when they had no regular visit to OB/GYNs. This implies that women who do not regularly see OB/GYNs, in particular, may be more likely to regard their PCPs as right people to ask about women’s health issues. More familiarity and established relationships with their PCPs or no distinctions or preferences between PCPs and OB/GYNs for women’s health issues as help-seeking sources might explain this phenomenon.

The study revealed that a relatively large proportion of women indicated high help-seeking intentions stemming from family/close friends and media. The information that women receive from these resources could influence their interpretations of women’s health issues and subsequent help-seeking behaviors. With family as a help-seeking source, PCPs need to take a family-oriented approach [26] to deepen an understanding of their help-seeking processes within a framework of the family system. For media, the study affirmed the previous finding that the internet is the main source of information used by women who are concerned about women’s health [27]. While media, especially the internet, is convenient and makes a wide range of health information accessible while simultaneously minimizing time spent and financial cost, not all information on the internet is trustworthy. PCPs need to be aware that women may present with ideas of women’s health issues filtered through information on the internet.

The meaning and extent of women’s concern about women’s health issues may vary from respondent to respondent, and their help-seeking intentions for PCPs may be influenced by unmeasured factors, such as their PCP’s scope of practice and the quality of the relationships between PCPs and their patients. Since obstetrics and gynecological care in family medicine are at a very early developmental stage in Japan and the study was conducted in primary care clinics where several PCPs actually practice gynecology and obstetrics, women may regard these family physicians as OB/GYNs, which may add a complexity in the interpretation of their help-seeking intentions. Additionally, we used the General Help Seeking Questionnaire to assess help-seeking intentions, but this questionnaire may not be able to capture all components of the conceptual framework of help-seeking behaviors: conscious planning, perception of effort to be exerted, willingness to communicate, perceptions of the problem, and perceived support, advice or assistance [28]. Help-seeking intentions may not necessarily predict actual help-seeking behaviors. Concerns about women’s health issues and help-seeking intentions could change over time and be affected by day-by-day events, and this cross-sectional study cannot grasp these dynamics. With such limitations, our study highlights the important implication that many women could present to a primary care clinic with unrevealed women’s health needs and that it is the PCP’s roles to enhance women’s health care.

The interpretation of the results requires caution. First, not having a regular visit to an OB/GYN does not necessarily indicate that they will not or are reluctant to see them for women’s health issues. Second, consecutive sampling from a limited number of primary care clinics during a short period of time in the study could lead to selection bias. The population in the study had higher rates of cervical and breast cancer screening than the national average (43.7 and 47.4%, respectively) [29]. Additionally, the proportion of the study participants who had high health literacy was greater than that of a general population in a previous study [30]. It is possible that they had a high level of health awareness and were more likely to have reported women’s health issues, leading to reporting bias [31]. Generalizability of the results could be limited due to sampling from primary care clinics, some of which a care in obstetrics and gynecology is actively provided, in the very restricted areas in Japan. Third, ethnic/racial minorities and women with intellectual challenges were not included. Transgender and nonbinary patients might not have been adequately addressed as participants. Therefore, health issues in those populations may not be well reflected in the results. Finally, the list of women’s health issues did not include intimate partner violence, perinatal mental health or substance abuse.

## Conclusions

A significant number of women visiting a primary care clinic had concerns about women’s health issues, and a majority of them had not regularly visited an OB/GYN. Women who do not regularly see OB/GYNs may be more willing to consult their PCPs about women’s health issues. PCPs need to be competent to handle unrevealed women’s health needs in daily practice.

## Data Availability

The datasets used and/or analyzed during the current study are available from the corresponding author on reasonable request.

## Abbreviations

PCPs: Primary care physicians
OB/GYNs: Obstetricians and gynecologists
